# Overlap of Scleroderma and Class V Lupus Nephritis: A Rare Autoimmune Confluence

**DOI:** 10.7759/cureus.56990

**Published:** 2024-03-26

**Authors:** Harshitha Reddy, Pranjal Kashiv, Sunil Kumar, Kapil N Sejpal, Sourya Acharya

**Affiliations:** 1 Internal Medicine, Jawaharlal Nehru Medical College, Wardha, IND; 2 Nephrology, Jawaharlal Nehru Medical College, Wardha, IND

**Keywords:** acute inflamm, membranous glomeluropathy, kidney disease, localized scleroderma, sytemic lupus erythematosus

## Abstract

The autoimmune connective tissue disease scleroderma is characterized by fibrosis of the skin, blood vessels, and visceral organs. Overlap syndromes are conditions in which a patient has characteristics from two or more autoimmune disorders. The coexistence of scleroderma and lupus nephritis is rare but documented. However, it is crucial to note that both scleroderma and lupus are complex autoimmune diseases with diverse clinical presentations and can affect multiple organ systems, including the kidneys. This case report presents a unique clinical scenario of a patient with the coexistence of scleroderma and systemic lupus erythematosus with Class V lupus nephritis, highlighting the challenges in diagnosis, treatment, and the need for a multidisciplinary approach.

## Introduction

Scleroderma comes from the Greek words *derma*, which means skin, and *scleros*, which means hardness. Skin fibrosis and thickening of blood vessels and internal organs are hallmarks of scleroderma [1]. It is divided into two subcategories: localized scleroderma and systemic sclerosis. Systemic sclerosis is distinct from localized scleroderma due to its association with Raynaud's phenomenon, acro-sclerosis, and involvement of internal organs. Kidney symptoms can develop as an initial manifestation of scleroderma without skin lesions.

Scleroderma presents with two distinct types of renal involvement. The initial type is scleroderma renal crisis (SRC) marked by a sudden onset of severe hypertension, retinopathy, and rapid decline in glomerular function resulting in reduced urine output and renal failure. The second type may manifest slowly. Symptoms encompass protein in urine, hematuria, and renal failure. Women are substantially more vulnerable with a frequency of between 50 and 300 cases per million people [2]. The fifth decade of life is the typical diagnostic age. It is linked to a greater death rate than other connective tissue disorders (CTDs) and can cause a significant reduction in quality of life. Systemic lupus erythematosus (SLE) is an autoimmune disease that affects multiple organ systems. The most prevalent of them is lupus nephritis, and its consequences are noteworthy because individuals with lupus nephritis may develop end-stage renal disease (ESRD) if they do not get treatment. An inflammatory response occurs in lupus nephritis, which triggers the complement system. Lupus nephritis is a hypersensitivity reaction (type 3) that causes immunological complex deposition on the glomerular basement membrane due to anti-dsDNA attachment to DNA, forming an anti-dsDNA [3,4]. Overlap syndrome typically happens in those with ribonucleoprotein antibody (RNP AB), but co-existence with the membranous Class V lupus nephritis variant is rare, and the pathophysiology is poorly understood [5].

## Case presentation

**Figure 1:**
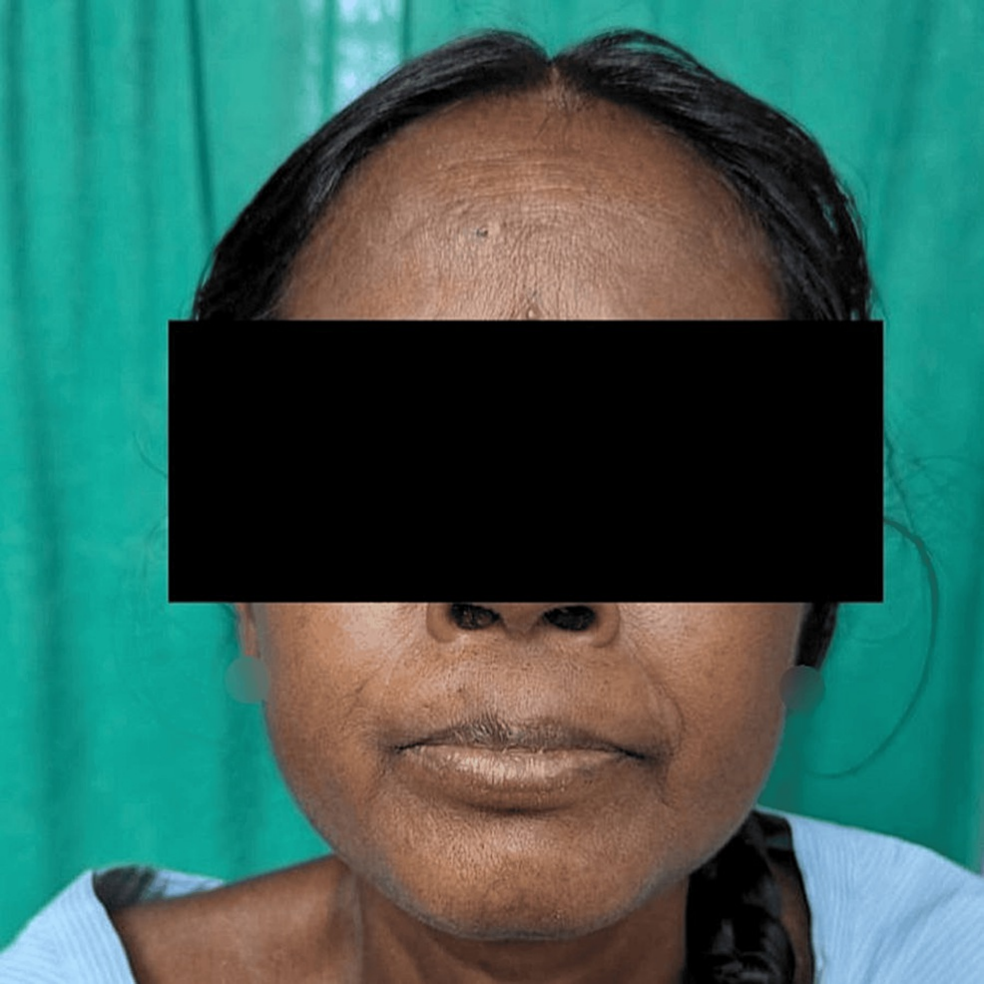
Thickened skin on the face

A 50-year-old female presented with complaints of progressively worsening bilateral upper and lower extremity joint pain, primarily involving fingers with early morning stiffness associated with diminished urine output for the past two days prior to hospitalization. She also had a progressive increase in bilateral lower limb edema. Physical examination revealed a blood pressure reading of 160/100 mmHg, pulse rate of 98 beats per minute, and respiratory rate of 14 cycles per minute. Saturation at room air is 98%. Her systemic examination was within normal limits. Thickening and hardening of the skin on the face were present (Figure 1).

All the laboratory parameters of the patient are highlighted in Table 1.

**Table 1:** Laboratory parameters of the patient ANA: Antinuclear antibody; CCP: Cyclic citrullinated peptide

Laboratory Parameters	Patient Value	Reference Range
Hemoglobin	5.8	13-15 g/dL
Total Leucocyte Count	45,800	4000-11000/mm^3^
Platelet Count	53,000	150,000-450,000/mm^3^
Mean Corpuscular Volume	80.5	79-100 fL
Urea	28	9-20 mg/dL
Creatinine	1.9	0.6-1.1 mg/dL
Sodium	139	137-146 mmol/L
Potassium	4.5	3.3-5.0 mmol/L
Alkaline Phosphatase	98	36-126 units/L
Alanine Transaminase	12	<50 units/L
Aspartate Transaminase	21	17-59 units/L
Total Protein	9.5	6.5-8.0 gm/dL
Albumin	3.0	3.4-5.0 gm/dL
Total Bilirubin	0.7	0.2-1.2 mg/dL
Globulin	6	2.3-3.5 gm/dL
Erythrocyte Sedimentation Rate	80	<15 mm/hr
ANA Titer	1:320	<1:80
Activated Partial Thromboplastin Clotting Time	30.1	29.5 control
Prothrombin Time	12.6	11.9 control
International Normalized Ratio	1.16	0.8-1.2
Spot Urine Protein Creatinine Ratio	2771	<200 mg/g
Anti-CCP	9.05	0-17 units/mL
C3 Complement	53.9	90-170 mg/dL
C4 Complement	14.3	10-40 mg/dL

**Figure 2:**
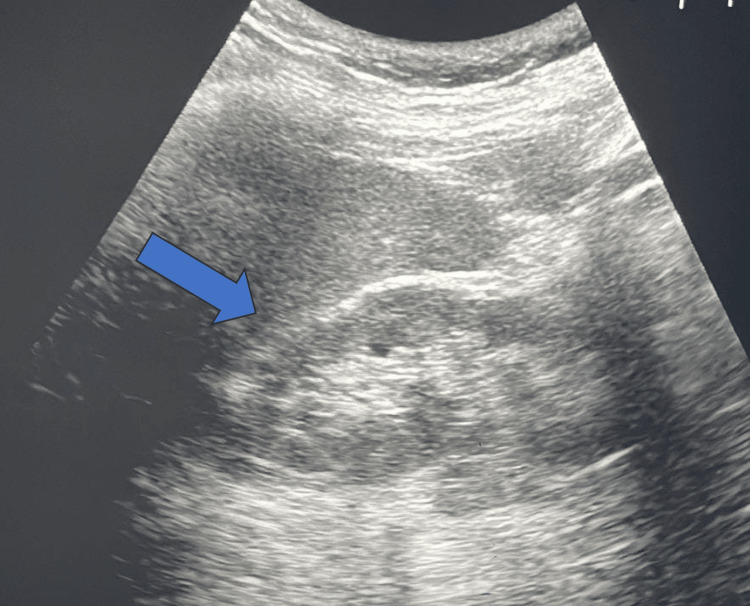
Ultrasonography of the abdomen was suggestive of normal shape, size, and echotexture of the kidneys (blue arrow)

On further investigation, anti-nuclear antibodies (ANA) and anti-scleroderma-70 antibodies were present. ANA profile was positive for ribonucleoprotein (nRNP) and anti-Smith. Ultrasonography of the abdomen was suggestive of normal shape, size, and echotexture of the kidneys (Figure 2).

**Figure 3:**
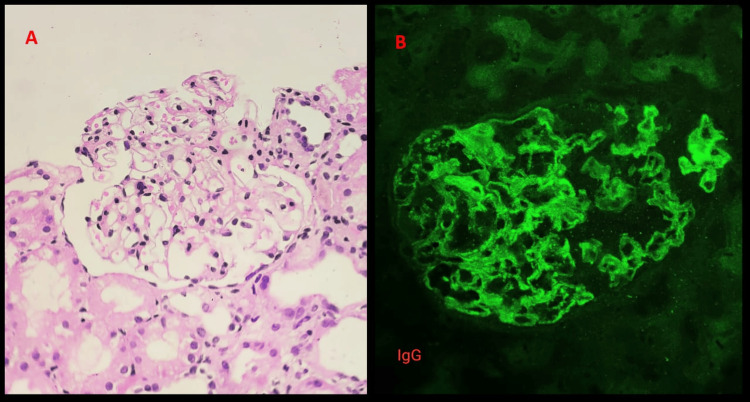
(A) Kidney biopsy showed a glomerulus with mild basement membrane thickening and patent capillary lumina. (B) Immunofluorescence microscopy-IgG showed global peripheral fine granular deposits of 3+ intensity along the glomerular basement membrane indicating subepithelial immune deposits.

Urine analysis revealed sub-nephrotic range proteinuria and kidney biopsy showed a glomerulus with mild basement membrane thickening and patent capillary lumina (Figure 3A). Immunofluorescence microscopy-IgG showed global peripheral fine granular deposits of 3+ intensity along the glomerular basement membrane indicating subepithelial immune deposits (Figure 3B).

Based on the biopsy findings, the patient was started on prednisolone and cyclophosphamide (modified Ponticelli regimen) and was on a monthly follow-up. In the follow-up, the protein in her urine was absent, indicating recovery.

## Discussion

Scleroderma and membranous lupus nephritis are autoimmune diseases characterized by systemic involvement and significant morbidity. The characteristic features of a particular connective tissue disease might overlap with those seen in another connective tissue disorder. Laboratory findings are not pathognomonic of any particular disease and should be identified clinically [6]. Overlap syndromes provide diagnostic and treatment challenges. This case report emphasises the uncommon association of scleroderma and membranous nephropathy, and how such a case should be diagnostically worked up or managed. The concept of shared autoimmunity describes the presence of autoantibodies circulating in healthy relatives of patients with acquired autoimmune diseases. This, along with the interplay of various environmental and genetic factors, leads to a myriad of clinical manifestations seen in such overlap syndromes [7].

Renal manifestations of scleroderma can occur in the absence of skin lesions and could be the earliest presenting symptom. Presentation could range from mild proteinuria and hematuria to marked hypertension and progressive and profound renal failure (scleroderma renal crisis), which is a medical emergency [8]. On the other hand, SLE commonly causes cutaneous and musculoskeletal manifestations but is very common to have some degree of renal manifestation ranging from Class I to Class VI nephritis. Class I is minimal mesangial lupus nephritis, Class II is mesangial proliferative lupus nephritis, Class III is focal lupus nephritis, Class IV is diffuse lupus nephritis, Class V is membranous lupus nephritis, and Class VI is advanced sclerosis lupus nephritis.

While most membranous nephropathy is primary (70-80%), there are other etiology such as drugs, infections such as hepatitis, malignancies, and autoimmune diseases such as lupus that can present with a membranous pattern of renal injury as the patient had [9]. This case is unique because not only the patient had a rare renal manifestation from scleroderma but also concurrently had a rare (Class V) renal manifestation of lupus nephritis.

The pathogenesis of scleroderma involves dysregulation of the immune system, leading to fibroblast activation and excessive collagen deposits. On the other hand, immunological complexes develop and deposit in the glomeruli of patients with membranous lupus nephritis, causing glomerular injury and initiating an inflammatory cascade. Scleroderma renal crisis is unique because while it can present with an acute kidney injury (AKI), angiotensin-converting enzyme inhibitors (ACEI) are usually the treatment of choice. Therefore, timely diagnosis and intervention are of paramount importance.

The membranous variant of lupus nephritis is also treated like primary membranous nephropathy with a modified Ponticelli regimen as the treatment of choice. This includes methylprednisolone (1gm) for three days then prednisolone (0.5 mg/kg/day) for the remaining 28 days, to be given at months one, three, and five, then cyclophosphamide (2-2.5mg/kg/day) for 30 days at months two, four, and six. Rituximab is often used as a second-line treatment when steroids are ineffective or side effects are intolerable.

Other second-line agents include tacrolimus, cyclosporine, chlorambucil, mycophenolate mofetil, and adrenocorticotropic hormone (ACTH) analogs. Patients with chronic kidney disease may benefit from a renal transplant [10].

## Conclusions

This case report sheds light on the complexity of autoimmune disorders as demonstrated by the coexistence of systemic sclerosis and membranous nephropathy. Additional research is required to understand the underlying pathophysiology of these coexisting autoimmune disorders. The diagnosis was based on the clinical criteria for systemic sclerosis and supported by specific autoantibodies, but the renal biopsy showed characteristic features of membranous nephropathy with immune complex deposition along the glomerular basement membrane. The overlapping presentation of these two autoimmune conditions posed a diagnostic challenge, necessitating careful consideration of clinical and laboratory findings.
